# Successful and Sustained Treatment of Cutaneous Tumoral Lesions in Brooke–Spiegler Syndrome (BSS) Using Ablative CO_2_ Laser: A Case Series and Literature Review

**DOI:** 10.1002/ccr3.70501

**Published:** 2025-05-08

**Authors:** Nikoo Mozafari, Mohammad Amin Jafari, Shirin Zaresharifi, Hassan Vahidnezhad, Leila Youssefian, Elham Behrangi, Farnoosh Seirafianpour, Arash Pour Mohammad, Alireza Jafarzadeh, Azadeh Goodarzi

**Affiliations:** ^1^ Skin Research Center Shahid Beheshti University of Medical Sciences Tehran Iran; ^2^ Department of Dermatology Loghman Hakim Hospital, Shahid Beheshti University of Medical Sciences Tehran Iran; ^3^ Department of Dermatology, Rasool Akram Medical Complex School of Medicine, Iran University of Medical Sciences (IUMS) Tehran Iran; ^4^ Center for Applied Genomics Children's Hospital of Philadelphia Philadelphia Pennsylvania USA; ^5^ Division of Human Genetics Children's Hospital of Philadelphia Philadelphia Pennsylvania USA; ^6^ Department of Pediatrics University of Pennsylvania, Perelman School of Medicine Philadelphia Pennsylvania USA; ^7^ Department of Pathology and Laboratory Medicine UCLA Los Angeles California USA; ^8^ Clinical Genomics Center David Geffen School of Medicine at UCLA Los Angeles California USA; ^9^ Razi Drug Research Center Iran University of Medical Sciences (IUMS) Tehran Iran; ^10^ Student Research Committee School of Medicine, Iran University of Medical Sciences Tehran Iran

**Keywords:** adnexal tumors, Brooke–Spiegler syndrome, CYLD, dermatology, trichoepitheliomas

## Abstract

Continuous‐wave CO_2_ laser therapy is an effective and safe treatment for trichoepitheliomas in Brooke–Spiegler syndrome, improving lesion color, texture, and overall appearance. This non‐invasive approach offers high patient satisfaction and demonstrated no recurrence during a 2‐year follow‐up, making it a promising alternative to surgical excision.

## 1 Introduction

Brooke–Spiegler syndrome (BSS) is a rare autosomal dominant condition characterized by multiple adnexal tumors, such as spiradenocylindroma, trichoepitheliomas, cylindromas, and spiradenomas, predominantly located in the head and neck area, with rare involvement in the trunk and extremities [1, 2, 3].

In 80%–85% of cases, BSS is attributed to a mutation in the CYLD gene, located on chromosome 16q12–q13. However, the penetrance and expression of this mutation vary, resulting in diverse clinical presentations and severity among individuals with the same germline mutation [4, 5]. Familial cylindromatosis, marked by multiple cylindromas [6], and multiple familial trichoepitheliomas, characterized by numerous trichoepitheliomas without other adnexal tumors [1], are also recognized as phenotypic variations of CYLD gene defects.

The predominant occurrence of adnexal tumors in the head and neck region [7] significantly impacts patients' appearance and poses a risk of malignant transformation in long‐standing lesions [8]. Therefore, developing an effective treatment modality for these patients is crucial. Various treatment approaches have been explored, including local excision as the standard treatment for individual lesions, Carbon dioxide (CO_2_) laser therapy, erbium: YAG laser therapy, electrosurgery, and cryosurgery [6, 7]. Additionally, different medical treatments, such as topical imiquimod, sodium salicylate, prostaglandin A1, and a combination of aspirin and adalimumab, have been proposed [9, 10, 11].

While surgery is typically considered the treatment of choice for trichoepitheliomas in BSS, it becomes impractical in cases with numerous lesions. Therefore, the CO_2_ laser, operating at a wavelength of 10,600 nm, is frequently employed for cutaneous lesions and skin resurfacing, offering a safe therapeutic alternative with excellent cosmetic outcomes and a low recurrence rate [12, 13, 14, 15, 16, 17].

This study introduces a novel application of continuous‐wave CO_2_ laser therapy tailored specifically for patients with BSS presenting multiple facial trichoepitheliomas. Unlike previous reports, our approach involved personalized laser parameter adjustment based on lesion size, independent post‐treatment photographic assessment, and a long‐term follow‐up of up to 2 years. To our knowledge, this combination of objective grading, patient‐reported outcomes, and extended observation has not been previously reported.

In this report, we present three family members all affected by BSS, with diagnoses confirmed through clinicopathological and genetic evaluations. Trichoepitheliomas of the face were biopsied and subsequently treated with CO_2_ laser ablation.

## 2 Case History/Examination

Three individuals from the same family, all diagnosed with Brooke syndrome confirmed through genetic testing and exhibiting multiple trichoepitheliomas on the central face, were enrolled in this study. Case 1 was a 34‐year‐old woman who presented with a cylindroma on the scalp and multiple facial trichoepitheliomas that first appeared at the age of 19. Case 2, the mother of the first case, was a 60‐year‐old woman who exhibited trichoepitheliomas on the face and cylindromas on the scalp, which first manifested when she was 22. Case 3, a 53‐year‐old man and the brother of the preceding case, had a 25‐year history of multiple trichoepitheliomas on the face and a few cylindromas on the scalp.

## 3 Methods

**FIGURE 1:**
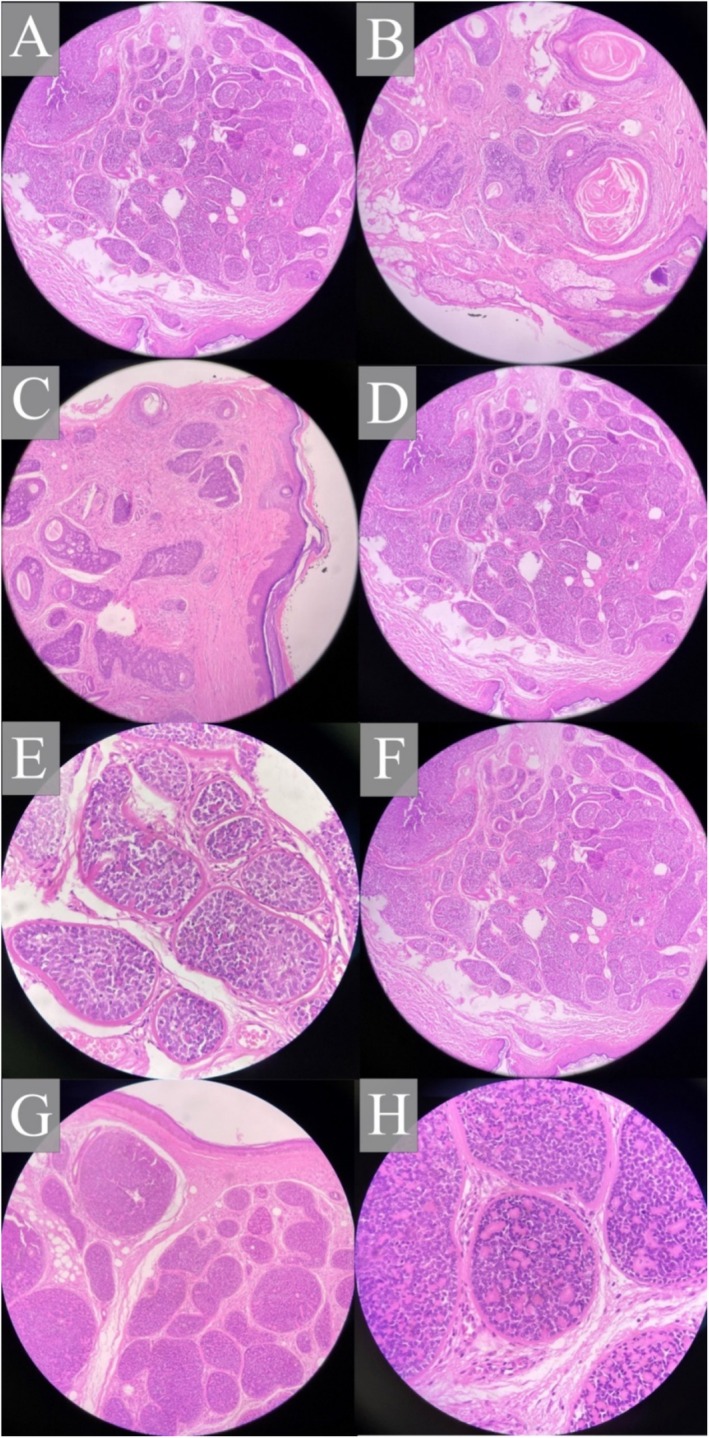
Histopathological views of trichoepitheliomas in Case 1 (A–C), Case 2 (D–F), and Case 3 (G, H).

Biopsies of selected lesions on the central face confirmed the presence of trichoepitheliomas. Histopathological analysis of these lesions revealed dermal tumors with fibrous stroma, various‐sized horn cysts, and basaloid epithelial structures (see Figure 1). Lesions were photographed using a digital camera under consistent lighting conditions at baseline, immediately post‐procedure, and at 2 months and 2 years following treatment.

The treatment was specifically targeted to the central facial region, including the nose, cheeks, and forehead, where the trichoepitheliomas were most pronounced. No laser therapy was applied to the scalp or other body areas affected by cylindromas.

Each patient underwent two sessions of continuous‐wave CO_2_ laser therapy (Edge One, Jeisys Medical Korea) for tumor ablation. Laser parameters included a frequency of 50 to 100 kHz, pulse duration of 500 to 800 μs depending on tumor size, power levels of 3 to 5 W, and 2 to 3 passes.

A board‐certified dermatologist, blinded to the treatment outcomes, evaluated and graded lesion photographs taken 2 months after treatment based on changes in pigmentation, texture, and overall improvement. The color of the lesions was scored from −5 (most hypopigmented) to +5 (most hyperpigmented). Texture was scored from 0 (most elevated) to 10 (flat). Overall improvement was scored from 0 (unchanged) to 10 (normal‐appearing skin).

Patients also rated their subjective satisfaction by indicating the percentage of improvement experienced after treatment sessions using a visual analogue scale ranging from 0 to 100.

Post‐treatment care included the application of topical antibiotic ointment, bland emollients, and strict sun protection using high‐SPF sunscreen. Patients were instructed to avoid sun exposure and to return for follow‐up visits at regular intervals. No systemic treatments or adjunctive therapies were administered post‐procedure.

## 4 Conclusion and Results

**FIGURE 2:**
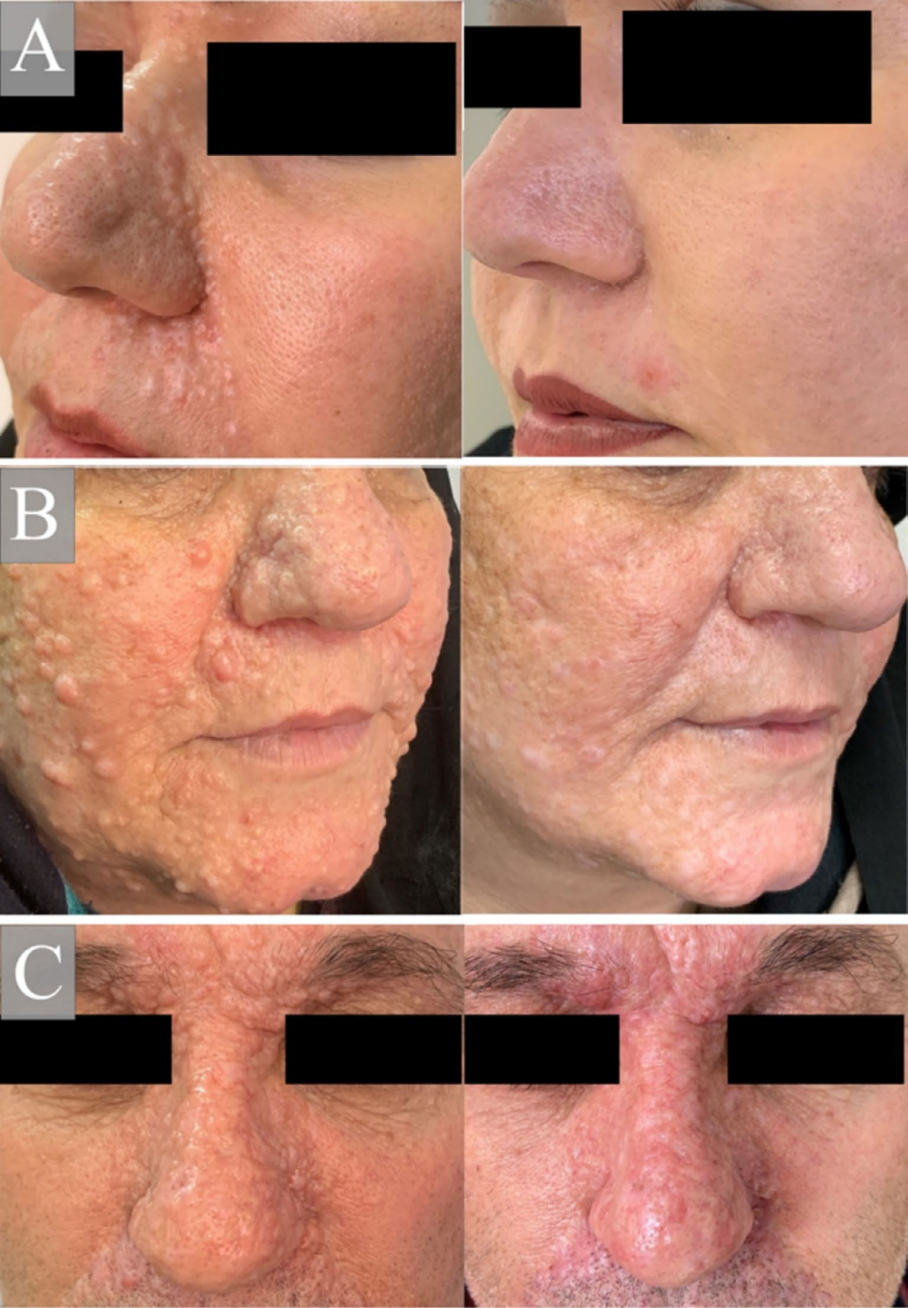
Photographs of trichoepithelioma lesions before and after treatment with two sessions of continuous‐wave CO_2_ laser therapy in Case 1 (A), Case 2 (B), and Case 3 (C).

Following treatment, nearly all lesions exhibited a flattened appearance and blended seamlessly with the surrounding healthy skin, indicating an excellent response to treatment. The overall dermatologists' score was 0 for all lesions prior to treatment. The mean texture score post‐treatment was 8. The treatment site displayed a coloration similar to or slightly lighter than the surrounding skin, possibly influenced by the family's medical history of vitiligo, which was managed with NBUVB therapy or topical treatments. The mean color score post‐treatment was −0.75. Additionally, the mean overall improvement score was 7.5 post‐treatment (see Figure 2). Patient satisfaction rates ranged from 75% to over 90%, with all patients expressing satisfaction with the procedure. Notably, there were no instances of recurrence during the 2‐ and 24‐month follow‐up periods.

## 5 Discussion

In this study, we treated three family members diagnosed with BSS, each presenting with numerous trichoepitheliomas on the central face, using continuous‐wave CO_2_ laser therapy sessions. Evaluations conducted 2 months post‐treatment revealed significant improvements in the color, texture, and overall appearance of all lesions. These findings highlight the potential of CO_2_ laser therapy as a promising treatment option for multiple trichoepitheliomas.

Previous studies have also investigated the efficacy of CO_2_ laser therapy in treating trichoepithelioma lesions, as outlined in Table 1.

**TABLE 1:** Summary of previous studies, which have used CO_2_ laser for Brooke–Spiegler syndrome along with trichoepitheliomas.

Author and year of publication	Case number	Gender/age	Lesion types	Location	Duration	Clinical features	Treatment	Results	Follow‐up/recurrence
Retamar et al. 2007 [12]	1	M/30	Multiple trichoepi	Central face and supraciliary regions	12 years	Asymptomatic, smooth‐surfaced, round, well‐demarcated, skin‐colored tumors	Continuous wave CO_2_ laser	Significant lesion reduction	NR
	2	F/38	Multiple trichoepi	Nasolabial folds	26 years	Round, firm, skin‐colored tumors	Continuous wave CO_2_ laser	Crusts for 2 weeks followed by slightly erythematous scars	NR
Martins et al. 2000 [13]	1	F/62	– Multiple trichoepi – Cylindromas	– Central face and nasolabial folds – Scalp	20 years	Rounded, firm, nontender, skin‐colored papules	Continuous wave CO_2_ laser	Good response	No recurrence after 1 year
	2	F/76	– Multiple trichoepi – Cylindromas	– Nasolabial folds – Entire scalp	20 years	Multiple dome‐shaped nodules, some ulcerated, others with telangiectasias	Continuous wave CO_2_ laser after e debulking of the larger cylindromas with bipolar scissors	Good response, areas of atrophy and alopecia in scalp	No recurrence after 1 year
Rallan et al. 2005 [14]	1	F/42	Multiple trichoepi	Forehead, nasolabial folds and upper lip	NR	Numerous skin‐colored papules	Combined erbium:Yag and CO_2_ laser for 3 sessions	Good cosmetic result	No recurrence after 2 years
Layegh et al. 2008 [16]	1	F/26	– Multiple trichoepi – Cylindromas	– Nasolabial folds, upper lip, and less commonly on the forehead and periocular areas – Scalp	12 years	Round‐to‐oval skin‐colored papules	Surgical excision for larger ones and CO_2_ laser for distributed ones	NR	NR
Peltonen et al. 2012 [18]	1	F/42	– Multiple trichoepi – Cylindromas – Spiradenomas	– Nose and eyebrows – Scalp – Chin	Since adolescence	NR	– CO_2_ laser – CO_2_ laser and surgical excision – Surgical excision	Scarring after laser treatment, cosmetically acceptable results after excision	No recurrence after excision until 2 years
Leventer et al. 2020 [17]	1	F/62	– Multiple trichoepi – Cylindromas – Spiradenomas	– Face – Scalp – Trunk	NR	Erythematous nodules on the scalp and trunk, skin‐colored papules on the face	CO_2_ laser for facial lesions and surgical treatment for others	NR	Constant recurrence
Tu et al. 2016 [19]	1	F/8	Multiple trichoepi	Paranasal, bilateral cheeks and medial canthi	4 years	Skin‐colored papules	Total face ablation with CO_2_ laser, followed by topical imiquimod and sirolimus	Pleasing results	Limited recurrence after adding topical sirolimus

Abbreviations: F, female; M, male; NR, not reported.

To our knowledge, seven prior studies have reported the treatment of trichoepitheliomas with laser therapy up to November 2024. Among these studies, a total of nine cases were identified as BSS accompanied by trichoepitheliomas, involving eight females and one male, with patient ages ranging from 6 to 76 years. With the exception of two studies that did not report final results [16, 17], all other studies [13, 14, 18, 19] demonstrated satisfactory outcomes following the treatment of lesions using continuous‐wave CO_2_ laser therapy. Furthermore, all studies confirmed no recurrence of lesions within a two‐year follow‐up period, except for the study by Leventer et al. [17].

Consistent with previous findings, our present study observed significant improvements in color, texture, and overall appearance among all patients, coupled with high levels of patient satisfaction. In our study, each patient underwent only two sessions of continuous‐wave CO_2_ laser therapy. However, some previous studies employed a combination of CO_2_ laser with other laser types such as Er:YAG [14, 20, 21] or topical treatments such as imiquimod and sirolimus [19]. While CO_2_ laser alone has produced good to excellent results, further comparative studies, particularly randomized clinical trials with larger sample sizes, are warranted to assess the effects of CO_2_ laser alone versus in combination with other treatment modalities.

Although our present study involved a larger sample size compared to previous studies, further increasing the sample size would provide more robust evidence for future treatments. Additionally, longer follow‐up periods should be considered to evaluate the recurrence rate of lesions post‐treatment, given the prolonged duration of most lesions in the absence of intervention.

## Author Contributions

**Nikoo Mozafari:** conceptualization, supervision. **Mohammad Amin Jafari:** writing – original draft. **Shirin Zaresharifi:** investigation, methodology. **Hassan Vahidnezhad:** data curation, validation, visualization. **Leila Youssefian:** investigation, methodology. **Elham Behrangi:** supervision, validation. **Farnoosh Seirafianpour:** writing – original draft. **Arash Pour Mohammad:** writing – review and editing. **Alireza Jafarzadeh:** writing – original draft, writing – review and editing. **Azadeh Goodarzi:** project administration, supervision.

## Disclosure

Transparency declaration: The authors affirm that the manuscript is honest, accurate, and transparent. No important aspect of the study has been omitted.

## Ethics Statement

Written informed consent was obtained from all patients, and their privacy was maintained in accordance with ethical standards. All patient photos included in this manuscript have been anonymized to ensure patient confidentiality. Based on the guidelines of the local medical research ethics committee, registration of this study is not required.

## Conflicts of Interest

The authors declare no conflicts of interest.

## Data Availability

The data that support the findings of this study are available from the corresponding author upon reasonable request.
